# Supplementary material for: Intra-slide calibration technology improves immunohistochemical harmonization within and between anatomic pathology laboratories
Source: bioRxiv. 2026 Jun 8:2026.06.04.730099. Preprint. [Version 1] doi: 10.64898/2026.06.04.730099 (PMC13278144; doi:10.64898/2026.06.04.730099)
Supplement: Supplementary file 1 [file media-1.pdf]

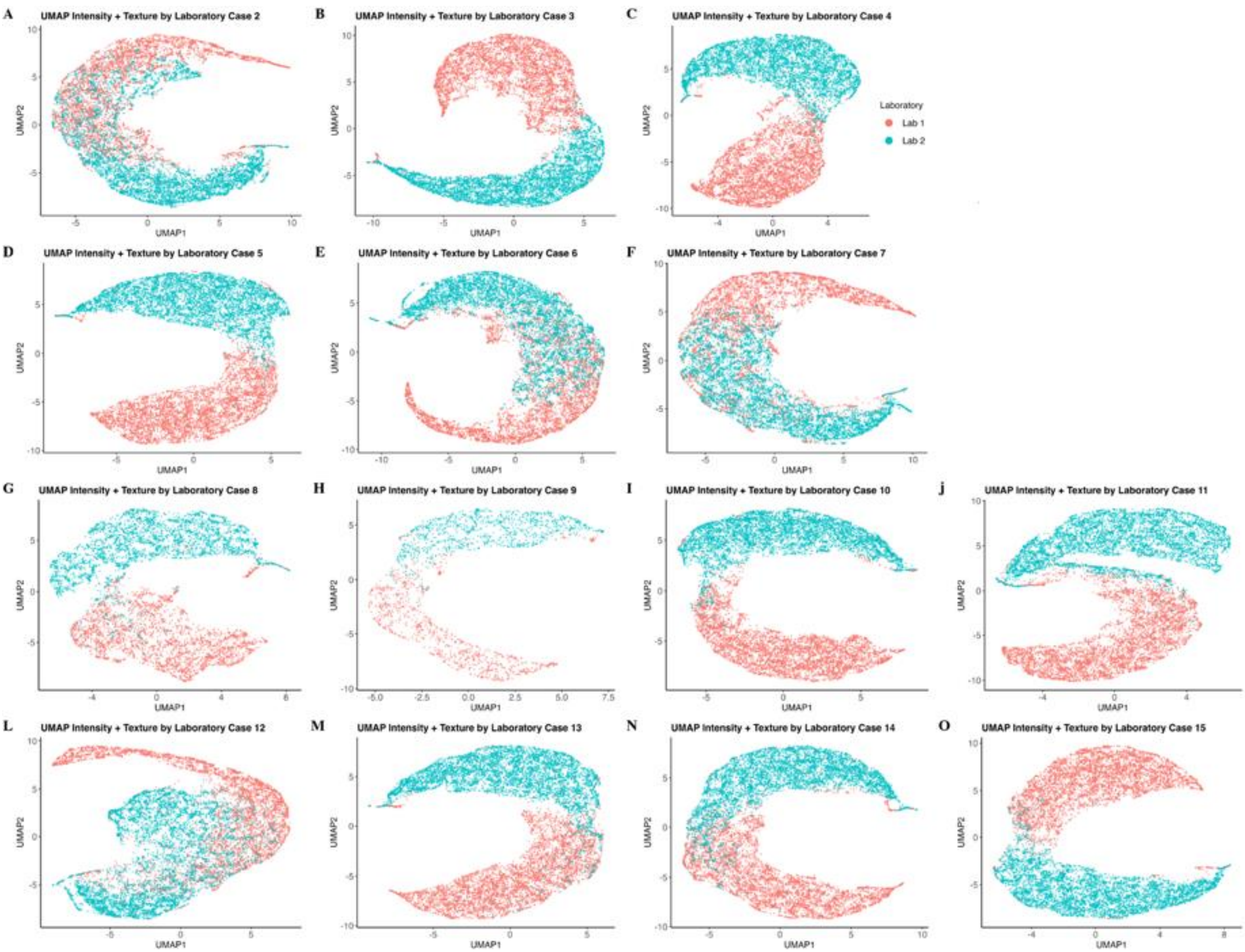

Supplementary Figure 1: Analysis of Inter-Laboratory Variability in Image Processing and Feature Extraction. (A-O) UMAP plot presents the distribution of intensity and texture features.

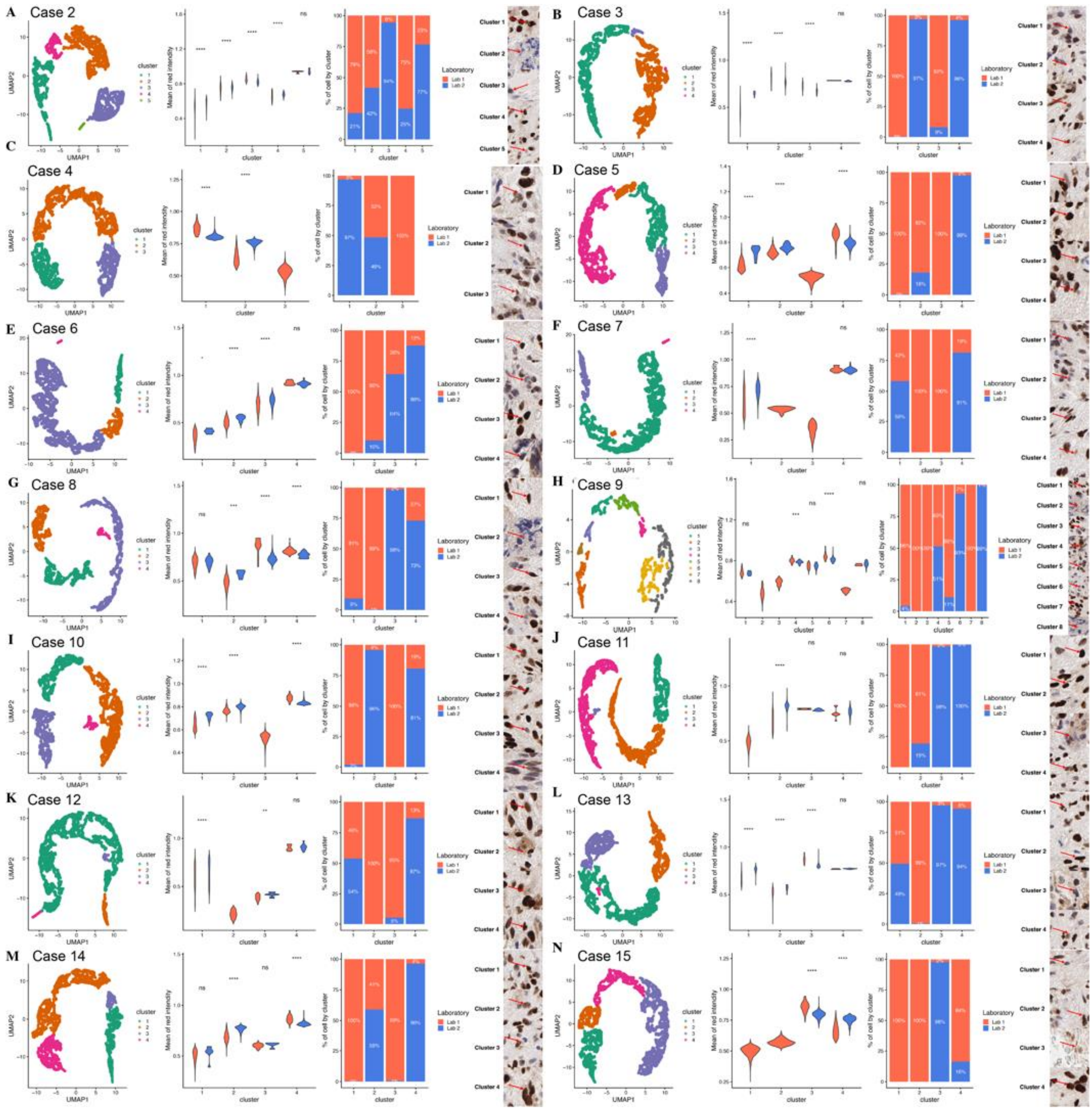

Supplementary Figure 2: Analysis of Tissue Cell Stanning for Inter-Laboratory Variability in Image Processing and Feature Extraction. (A-N) UMAP plot presents the DBscan cluster distribution of intensity feature, on the left showing the cluster distributions, violin plots in the

middle displaying the intensity differences between clusters, barplots showing the percentage of cell distributed in each cluster and representative cell images on the right for each cluster.

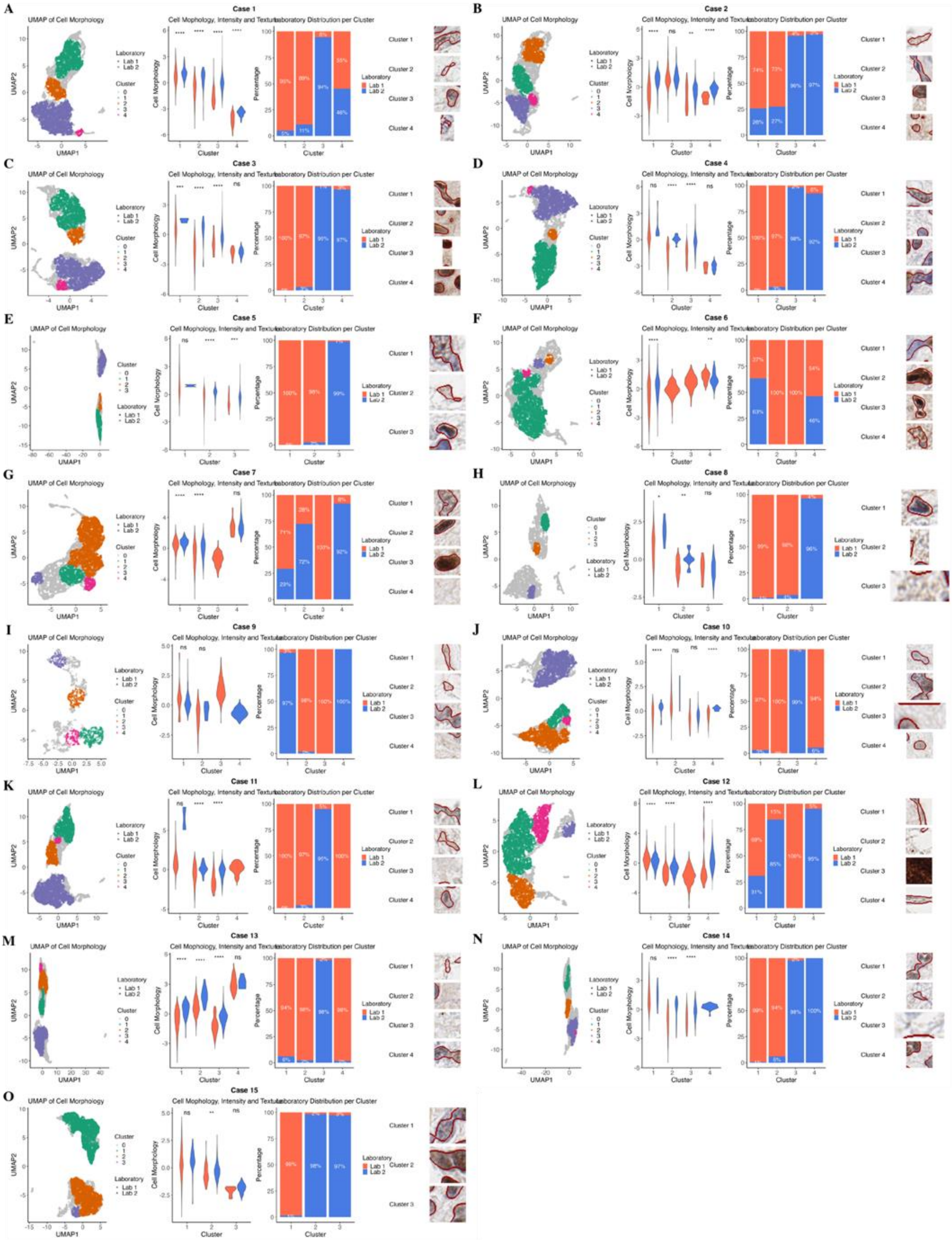

A
Case 1
UMAP of Cell Morphology
Cell Mophology, Intensity and Texture
Laboratory Distribution per Cluster
B
Case 2
C
Case 3
D
Case 4
E
Case 5
F
Case 6
G
Case 7
H
Case 8
I
Case 9
J
Case 10
K
Case 11
L
Case 12
M
Case 13
N
Case 14
O
Case 15
UMAP1
UMAP2
Cell Morphology
Percentage
Cluster
Laboratory
Lab 1
Lab 2
Cluster 1
Cluster 2
Cluster 3
Cluster 4

Supplementary Figure 3: Analysis of Tissue Cell Stanning for Inter-Laboratory Variability in Image Processing and Feature Extraction. (A-N) UMAP plot presents the DBscan cluster distribution of morphology: intensity, texture, shape, position and density features, on the left showing the cluster distributions, violin plots in the middle displaying the morphology differences between clusters, barplots showing the percentage of cell distributed in each cluster and representative cell images on the right for each cluster.

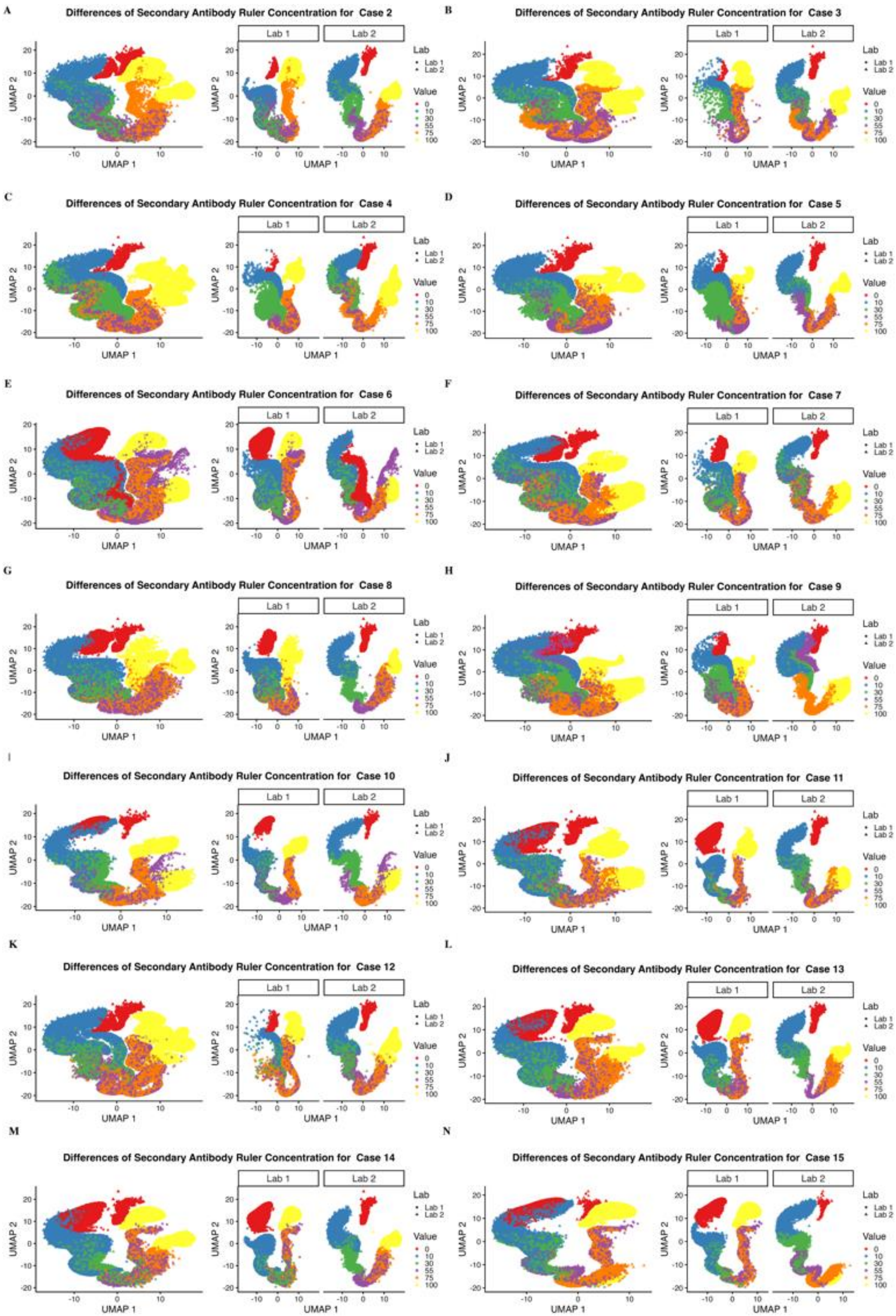
A
Differences of Secondary Antibody Ruler Concentration for Case 2
B
Differences of Secondary Antibody Ruler Concentration for Case 3
C
Differences of Secondary Antibody Ruler Concentration for Case 4
D
Differences of Secondary Antibody Ruler Concentration for Case 5
E
Differences of Secondary Antibody Ruler Concentration for Case 6
F
Differences of Secondary Antibody Ruler Concentration for Case 7
G
Differences of Secondary Antibody Ruler Concentration for Case 8
H
Differences of Secondary Antibody Ruler Concentration for Case 9
I
Differences of Secondary Antibody Ruler Concentration for Case 10
J
Differences of Secondary Antibody Ruler Concentration for Case 11
K
Differences of Secondary Antibody Ruler Concentration for Case 12
L
Differences of Secondary Antibody Ruler Concentration for Case 13
M
Differences of Secondary Antibody Ruler Concentration for Case 14
N
Differences of Secondary Antibody Ruler Concentration for Case 15
Lab 1
Lab 2
UMAP 1
UMAP 2
Lab
Value
0
10
30
55
75
100

Supplementary Figure 4: Analysis of Secondary Antibody Concentration Ruler for Inter-Laboratory Variability in Image Processing and Feature Extraction. (A–N) UMAPs showing differences in the distribution of each secondary antibody concentration across laboratories for each case.

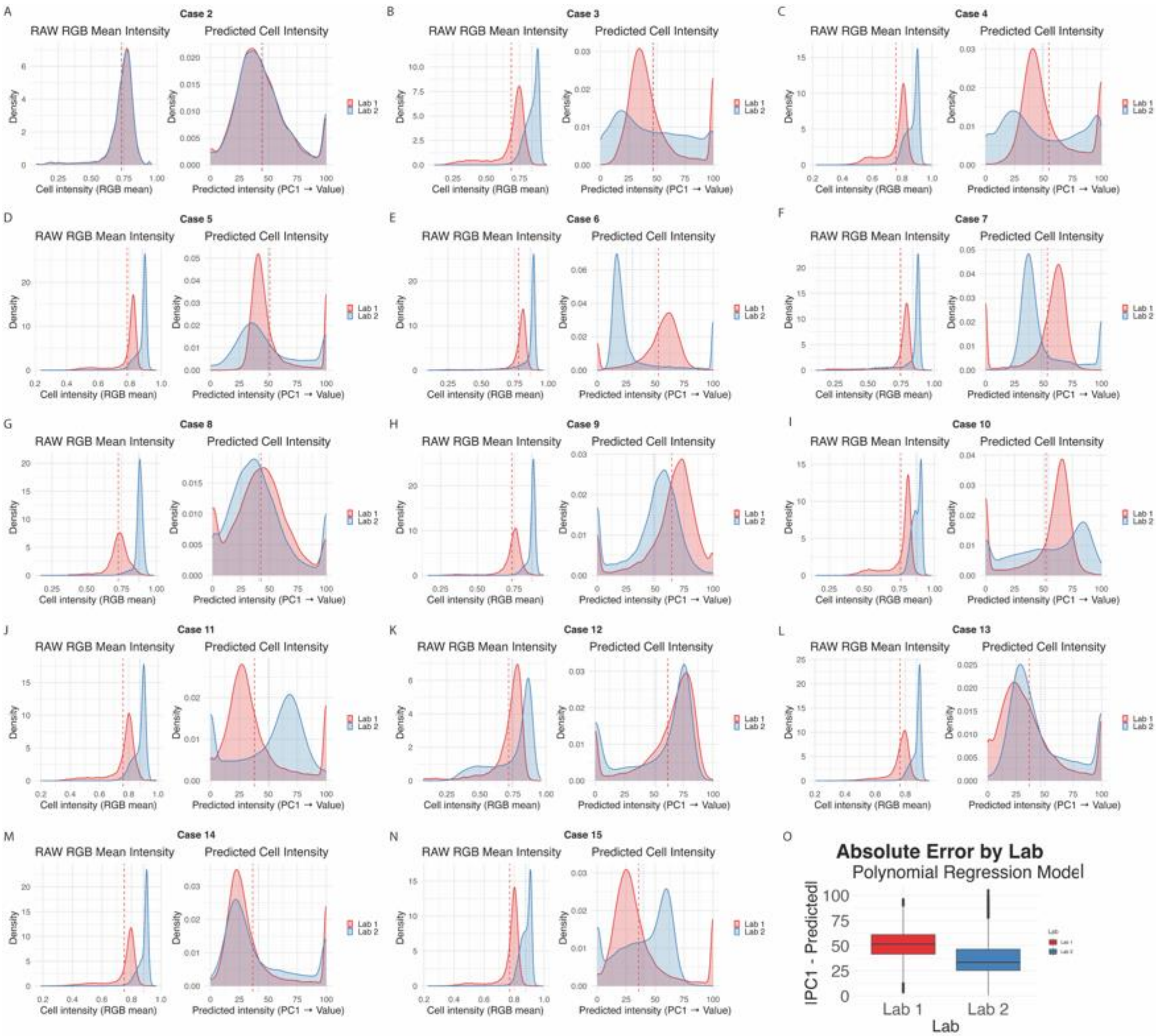

Supplementary Figure 5. Predictive model for quantifying p53 antibody cell intensity between two laboratories (Lab 1 and Lab 2). (A-N) Histogram from Cases demonstrating the harmonization effect. (O) The boxplots display Comparison of measurement distributions.

Supplementary Table 1: Percent reduction of the per-case inter-laboratory calibration technology Δ|SMD| before and after harmonization,

Case	***|SMD| RAW***	***|SMD| PRED***	$\Delta\|SMD\|$	***% reduction***
Case_1	2.12883576	0.16119591	1.96763986	92.4279783
Case_2	0.00224944	0.07791183	-0.0756624	NA
Case_3	1.7402548	0.1723174	1.5679374	90.0981511
Case_4	1.65998441	0.28504992	1.37493449	82.8281566
Case_5	1.56100372	0.18856122	1.3724425	87.9205144
Case_6	1.12046263	0.12972931	0.99073331	88.421808
Case_7	0.88537675	0.06775231	0.81762443	92.3476289
Case_8	2.63437984	0.11006772	2.52431212	95.8218735
Case_9	2.33827232	0.40614445	1.93212787	82.6305754
Case_10	1.64714308	0.12796529	1.51917779	92.231076
Case_11	1.58492265	0.18065309	1.40426956	88.6017726
Case_12	0.17557956	0.19922754	-0.023648	-13.468526
Case_13	2.01893629	0.28527256	1.73366373	85.8701556
Case_14	1.90642958	0.10431353	1.80211605	94.5283305
Case_15	1.77204265	0.07314456	1.69889808	95.8723025
Total	1.66	0.16	1.5	90.3

RAW: PC1 before prediction in pixels units; PRED: predicted after calibrated in %; |SMD|: Standard Mean Deviation; Δ|SMD| $= (RAW - PRED)$; NA: data < 0,10 threshold.
